# Longitudinal assessment of motor function following the unilateral intrastriatal 6-hydroxydopamine lesion model in mice

**DOI:** 10.3389/fnbeh.2022.982218

**Published:** 2022-11-24

**Authors:** Xiuping Sun, Xianglei Li, Ling Zhang, Yu Zhang, Xiaolong Qi, Siyuan Wang, Chuan Qin

**Affiliations:** ^1^National Health Commission Key Laboratory of Human Disease Comparative Medicine, Beijing Engineering Research Center for Experimental Animal Models of Human Critical Diseases, National Center of Technology Innovation for Animal Model, Institute of Laboratory Animal Sciences, Chinese Academy of Medical Sciences (CAMS) & Peking Union Medical College (PUMC), Beijing, China; ^2^Changping National Laboratory (CPNL), Beijing, China

**Keywords:** Parkinson’s disease, 6-OHDA, mice, gait analysis, cylinder test, rotarod test, apomorphine-induced rotational test

## Abstract

**Introduction:**

Despite the widespread use of the unilateral striatal 6-hydroxydopamine (6-OHDA) lesion model in mice in recent years, the stability of behavioral deficits in the 6-OHDA striatal mouse model over time is not yet clear, raising concerns about using this model to evaluate a compound’s long-term therapeutic effects.

**Materials and methods:**

In the current study, mice were tested at regular intervals in the cylinder test and gait analysis beginning 3 days after 6-OHDA injection of 4 and 8 μg and lasting until 56 days post-lesion. Apomorphine-induced rotational test and rotarod test were also performed on Day 23 and 43 post-lesion, respectively. Immunohistochemistry for dopaminergic neurons stained by tyrosine hydroxylase (TH) was also performed.

**Results:**

Our results showed that both the 4 and 8 μg 6-OHDA lesion groups exhibited forelimb use asymmetry with a preference for the ipsilateral (injection) side on Day 3 and until Day 21 post-lesion, but did not show forelimb asymmetry on Day 28 to 56 post-lesion. The 8 μg 6-OHDA lesion group still exhibited forelimb asymmetry on Day 28 and 42 post-lesion, but not on Day 56. The gait analysis showed that the contralateral front and hind step cycles increased from Day 3 to 42 post-lesion and recovered on Day 56 post-lesion. In addition, our results displayed a dose-dependent reduction in TH^+^ cells and TH^+^ fibers, as well as dose-dependent apomorphine-induced rotations. In the rotarod test, the 8 μg 6-OHDA lesion group, but not the 4 μg group, decreased the latency to fall on the rotarod on Day 43 post-lesion.

**Conclusion:**

In summary, unilateral striatal 6-OHDA injections of 4 and 8 μg induced spontaneous motor impairment in mice, which partially recovered starting on Day 28 post-lesion. Forced motor deficits were observed in the 8 g 6-OHDA lesion group, which remained stable on Day 43 post-lesion. In addition, the rotarod test and apomorphine-induced rotational test can distinguish between lesions of different extents and are useful tools for the assessment of functional recovery in studies screening novel potential therapies.

## Introduction

Parkinson’s disease (PD) is a progressive neurodegenerative disorder that causes motor disturbances such as rigidity, resting tremor, bradykinesia, and postural instability (Tolosa et al., 2006; Coelho and Ferreira, 2012; Kalia and Lang, 2015). Experimental evidence shows that the motor impairment of PD results from the degeneration of dopaminergic neurons in the substantia nigra (SN) *pars compacta*, which results in the loss of nerve terminals in the striatum (Dauer and Przedborski, 2003; Drui et al., 2014). Unilateral onset and persistent asymmetry of motor deficits are important characteristics of PD (Ham et al., 2015; Miller-Patterson et al., 2018). In addition, PD causes nonmotor symptoms such as hyposmia, sleep disorders, depression, and constipation, which are sometimes present before diagnosis and almost always emerge with disease progression (Chaudhuri et al., 2006; Schapira et al., 2017).

The unilateral 6-hydroxydopamine (6-OHDA) lesion model has been widely used to produce side-bias motor deficits that reflect the asymmetric motor symptoms seen in patients with PD (Lizarraga et al., 2017; Fiorenzato et al., 2021). Motor deficits caused by 6-OHDA lesions of SN neurons have been well characterized in rats (Lee et al., 1996; Yuan et al., 2005; Su et al., 2018). A growing body of evidence has shown that behavioral impairment in mice has also been observed (Glajch et al., 2012; Smith et al., 2012; Park et al., 2015). Grealish et al. (2010) reported that the corridor task and apomorphine-induced rotational test can be used to predict the extent of dopaminergic lesions in the intranigral 6-OHDA lesion model in mice. Iancu et al. (2005) reported that the amphetamine-induced rotation test, the cylinder test, and the rotarod test were the most sensitive and reliable in detecting the loss of dopaminergic cells in the SN. Glajch et al. (2012) observed that the cylinder test, challenging beam, pole test, adjusting steps, and spontaneous activity tests are all highly robust assays for detecting sensorimotor impairments in the 6-OHDA mouse model. The severity of symptoms in the 6-OHDA model is determined by the site of injection and the dose of 6-OHDA used (Grealish et al., 2010; Park et al., 2015). Unilateral injection of 6-OHDA into the medial forebrain bundle (MFB) causes an acute and almost complete loss of dopamine neurons in the SN (Glajch et al., 2012). In contrast, intrastriatal administration of 6-OHDA results in a chronic and partial loss of SN DA neurons, similar to what is seen in patients with PD (Yuan et al., 2005; Alvarez-Fischer et al., 2008; Bagga et al., 2015). Bagga et al. (2015) found that striatal lesions were more reliable, with a strong correlation between dopamine neuron loss and rotational asymmetry compared to MFB lesions. Lee et al. (1996) reported that striatal 6-OHDA lesions produce dose-dependent decreases in striatal dopamine levels and dopaminergic cell numbers in the ipsilateral SN. Furthermore, the time course of behavioral changes and associated pathological features of the 6-OHDA lesion model in rats has been described (Su et al., 2018). Hsieh et al. (2011) observed that gait disturbances occurred as early as 4 days post-lesion and gradually increased up to 42 days post-MFB lesions. Sauer and Oertel (1994) reported that the injection of 6-OHDA into the terminal field of nigral dopaminergic neurons caused progressive degeneration of these cells, starting between 1 and 2 weeks after the lesion and lasting 8–16 weeks. Moreover, spontaneous recovery of dopaminergic fibers and terminals in the dorsal striatum has been observed in rats 16 weeks post-SN lesions (Stanic et al., 2003), raising concerns about the suitability of this model for the assessment of long-term therapeutic effects of a compound. Some studies have also characterized the time course of intrastriatal 6-OHDA lesions in mice (Park et al., 2015). Grealish et al. (2010) investigated dopamine neuron loss at several different time points after the intrastriatal delivery of 6-OHDA (3 and 6 h, and 1, 3, 6, 9, and 12 days) and found that, despite rapid loss of TH^+^ fibers in the striatum, the reduction in the number of TH^+^ neurons in the SN was delayed. Alvarez-Fischer et al. (2008) found that striatal injections of 6-OHDA induced a stable loss of nigral dopaminergic neurons and a behavioral impairment that was partially reversible in rotarod motor performance over a 2-month time course.

To date, no detailed description of longitudinal behavioral characterization in mice has been published. The long-term stability of behavioral deficits in the 6-OHDA striatal mouse model is unknown. In this study, a battery of behavioral tests was used to identify behavioral characteristics that were significantly changed in the hydroxydopamine mouse model of PD. The stability of motor deficits over time was studied. Mice were tested in the cylinder test and gait analysis at regular intervals beginning 3 days after lesioning and lasting 56 days. Apomorphine-induced rotational tests and rotarod tests were also performed on Day 23 and 43 post-lesion, respectively. TH immunostaining was also performed.

## Materials and methods

### Animals

A total of 33 male C57BL/6J mice (from Beijing HFK Bioscience Co., Ltd., 10 weeks old at the time of the surgery) were used in this study. All animals were housed 3–5 per cage under standard conditions (22–23°C with a 12/12-h light/dark cycle) with free access to food and tap water. All the experimental procedures were conducted in compliance with the guidelines for the ethical review of laboratory animal welfare, People’s Republic of China National Standard GB/T 35892-2018 (MacArthur Clark and Sun, 2020), and were approved by the Institutional Animal Care and Use Committee of the Institute of Laboratory Animal Sciences (No: QC21002). The experiment was performed on 20 April 2021.

### Surgical procedure

6-Hydroxydopamine (Sigma, USA) was dissolved in a solution of 0.2 mg/ml of ascorbic acid in 0.9% NaCl. The mice were divided randomly into the sham group (*n* = 10), the 4 μg 6-OHDA group (*n* = 11), and the 8 μg 6-OHDA group (*n* = 12). The mice were anesthetized with isoflurane (1.5%, inhaled) and mounted in a stereotaxic frame with a mouse adaptor (RWD Life Science, Shenzhen, China). The 4 and 8 μg 6-OHDA groups received an injection of 2 μl of ascorbic acid in 0.9% NaCl with 6-OHDA (free base) at a concentration of 2 or 4 mg/ml into the left striatum. The SHAM group received an injection of the vehicle solution (0.2 mg/ml ascorbic acid in 0.9% NaCl). 6-OHDA (4 and 8 μg) was injected using a Hamilton syringe at two different sites of the left striatum with 1 μl each at a rate of 0.5 μl/min. Lesion coordinates (relative to bregma) were (i) AP = +1.0 mm, ML = 2.1 mm, DV = −2.9 mm relative to Bregma and (ii) AP = +0.3 mm, ML = 2.3 mm, DV = −2.9 mm (corresponding to the atlas of Paxinos and Franklin, 1997). The syringe was left in place for 5 min before it was slowly drawn back. The skin was then sutured. The mice received 5 mg/kg meloxicam SC as an analgesic and were allowed to recover before being returned to their home cage.

### Behavioral tests

**FIGURE 1:**
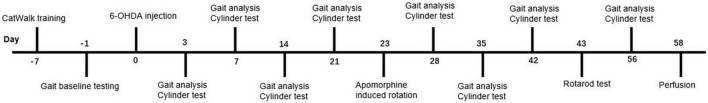
Time course of the experiment. CatWalk XT training and baseline testing of gait analysis were conducted in the week preceding the 6-OHDA lesion. Gait analysis and cylinder test were performed at day 3, 7, 14, 21, 28, 35, 42, and 56 post lesion on the same day. Apomorphine-induced rotation and rotarod test were performed at day 23 and day 43 post lesion. All animals were sacrificed after behavioral testing.

Thirty-three male C57BL/6J mice were used for gait analysis, cylinder test, apomorphine-induced rotational test, and rotarod test. The animals were divided randomly into the sham group (*n* = 10), the 4 μg 6-OHDA group (*n* = 11), and the 8 μg 6-OHDA group (*n* = 12). Behavioral tests were performed during the light phase of the light/dark cycle and in temperature- and humidity-controlled rooms. On the day of the experiment, mice were moved to the testing room, where they remained in their home cage for a 60 min acclimation period. Gait analysis was performed and preceded the cylinder test on the same day. Rotarod training was performed after routine gait analysis/cylinder testing. All tests were performed at different time points post-lesion over a period of 56 days (Figure 1).

#### Gait analysis

The gait of naturally moving mice was analyzed with CatWalk XT (Noldus Information Technology, Netherlands) as previously described (Kappos et al., 2017; Garrick et al., 2021). The CatWalk gait system consists of a glass walkway floor illuminated with a green light that is completely internally reflected in the glass, a standard charge-coupled device (CCD) camera underneath, and software for recording and analyzing the data obtained from mouse paws. A goal box was used at the end of the walkway to create an incentive to cross the field, where the entrance to the mouse home cage is placed. Before 6-OHDA lesioning, all mice were first placed in the front of the start zone of the walkway and were trained to cross the walkway with five accomplished runs per day on six consecutive days. A successful run was defined as a mouse walking across the runway without any hesitation. Baseline testing was conducted before surgery. Gait analysis began on Days 3, 7, 14, 21, 28, 35, 42, and 56 after 6-OHDA lesioning and consisted of three runs. The walkway was cleaned thoroughly with 70% ethanol between each animal. The following parameters were evaluated: average speed, cadence, step cycle, stand and swing duration, print area, and print width. Gait parameters of footprints were automatically generated (left front, LF; left hind, LH; right front, RF; and right hind, RH).

#### Cylinder test

Forelimb use asymmetry during explorative activity was analyzed in the cylinder test according to the protocol described previously (Zhu et al., 2018). Mice were individually placed in a plexiglass cylinder (diameter: 9.5 cm, height: 20 cm) and immediately videotaped for 3 min. Two mirrors were placed behind the cylinder at an angle to record the mice from all sides. The videos were analyzed in slow motion by an investigator blinded to the experiment. Wall contacts with the ipsilateral forelimb, contralateral forelimb, or both forelimbs on the wall of the cylinder were counted. The percentage of ipsilateral contacts relative to the total number of contacts was calculated. The cylinder test began on Days 3, 7, 14, 21, 28, 35, 42, and 56 after 6-OHDA lesioning.

#### Apomorphine-induced rotational test

On Day 23 post-lesion, the apomorphine-induced rotational test was performed to evaluate the severity of striatal lesioning in mice. Each mouse was placed in a mouse cage (26 × 16 × 12 cm) and was allowed to habituate to the environment for 10 min before the rotation test. Mice were given 0.5 mg/kg apomorphine intraperitoneally (dissolved in 0.2 mg/ml ascorbic acid in 0.9% saline solution) and then returned to their cages. Full 360° turns contralateral to the lesion were counted manually for 30 min using a stopwatch. The data were analyzed to determine the number of contralateral rotations (Smith et al., 2012).

#### Rotarod test

In the training phase of the rotarod test, mice underwent three trials per day on three consecutive days (Days 40, 41, and 42 post-lesion). The speed of the rotation was increased gradually from 5 to 24 rpm during the first 40 s and then held constant at that rate for the rest of the trial (260 s). During the test phase (Day 43 post-lesion), the speed of the rod increased from 4 to 40 rpm for 300 s. Mice were scored for their latency to fall.

### Tissue processing and immunohistochemistry

After the behavioral tests were completed, 33 mice were terminally anesthetized with sodium pentobarbital (100 mg/kg body weight, i.p.) and transcardially perfused with 0.9% NaCl followed by ice-cold 4% paraformaldehyde (PFA) in phosphate-buffered saline. Brains were post-fixed for 24 h in 4% PFA before being transferred to 20% sucrose in 0.1 M PBS for cryoprotection. Immunohistochemistry was performed on free-floating 50 m coronal sections of the entire striatum and midbrain. Selected sections were then incubated overnight with anti-tyrosine hydroxylase (TH) antibody at a concentration of 1:5,000 (ab137869, Abcam). Immunoreactions were revealed by an anti-species dependent peroxidase Envision™ system (DAKO) followed by DAB visualization. Sections were mounted on gelatinized slides, counterstained with 0.1% cresyl violet solution if needed, dehydrated in alcohol, cleared in xylene, and cover-slipped with DPX. As our previous study described, the number of neurons in the SN was counted by stereology using a Leica DM-6000B microscope coupled with Mercator Pro software (Explora Nova) (Sun et al., 2021). A total of five sections of SN, between 2.5 and 4.1 relative to Bregma, were counted per animal. TH^+^ neurons in the SN were estimated according to the optical fractionator principle. The TH staining in the striatum was quantified by optical density (OD). Sections were scanned with a high-resolution Epson Expression 10000XL scanner. The same parameters were applied to and analyzed in all sections. Data are expressed as the percentage of immunostaining intensity on the lesioned side over the non-lesioned side.

### Statistical analyses

SPSS 20 or GraphPad Prism 8 software was used for statistical analysis. All data are presented as the mean ± standard error of the mean (SEM). A linear mixed model was used for the statistical evaluation of weight, gait analysis, forelimb use asymmetry, and latency to fall in the rotarod test with time as a repeated measure, followed by Bonferroni’s multiple comparisons test where appropriate. Average speed was added as a covariate in the gait analysis of dynamic paw parameters, such as step cycle, stand phase, swing phase, print position, and cadence, considering that these parameters have been reported previously to correlate with speed. The influence of time, treatment, or time × treatment on the different parameters was evaluated. Statistical significance for rotation testing was analyzed using one-way ANOVA with Bonferroni’s *post hoc* test. The significance level was set at *P* < 0.05.

## Results

1. Unilateral injection of 4 and 8 μg 6-OHDA did not affect body weight or cause death in mice.

**FIGURE 2:**
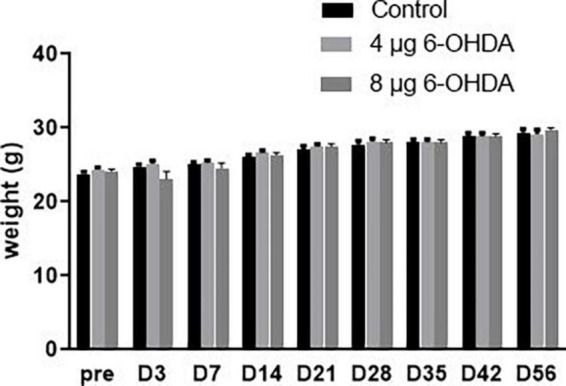
Neither 4-μg nor 8-μg 6-OHDA lesion group affected the mice body weight. All results are presented as mean ± standard error of the mean (SEM).

No significant difference was observed in body weight for either the 4 or 8 μg 6-OHDA lesion groups when compared to the sham group. There were no premature deaths before the end of the test (Figure 2).

2. Unilateral injection of 4 and 8 μg 6-OHDA induced forelimb use asymmetry in mice.

**FIGURE 3:**
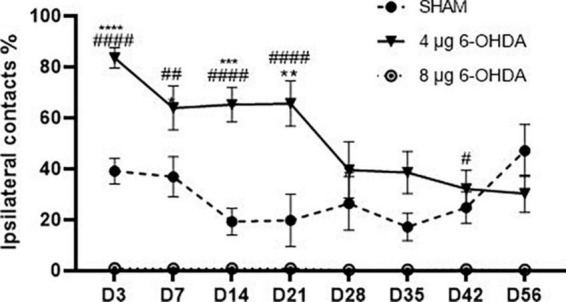
Time-course changes in the percentage of ipsilateral contacts in the cylinder test following 4-μg and 8-μg 6-OHDA injection. All results are presented as mean ± standard error of the mean (SEM). ****P < 0.0001, ***P < 0.001, *P < 0.05, SHAM vs. 4-μg lesion group; ^####^*P* < 0.0001, ^#^*P* < 0.05, SHAM vs. 8-μg lesion group.

A significant effect of the group × time interaction (*F*_(14,199)_ = 3.65, *P* < 0.0001) was found on the percentage of ipsilateral contacts. *Post hoc* multiple comparisons showed that the percentage of ipsilateral contacts was higher in the 4 and 8 μg 6-OHDA lesion groups than in the sham group on Days 3, 7, 14, and 21 post-lesion, and a significant difference was observed in the 8 μg 6-OHDA lesion group compared with the sham group on Days 28 and 42 post-lesion. There was no significant difference between the 4 and 8 μg lesion groups (Figure 3).

3. Unilateral injection of 4 and 8 μg 6-OHDA induced gait pattern abnormalities in mice.

*Average speed*

**FIGURE 4:**
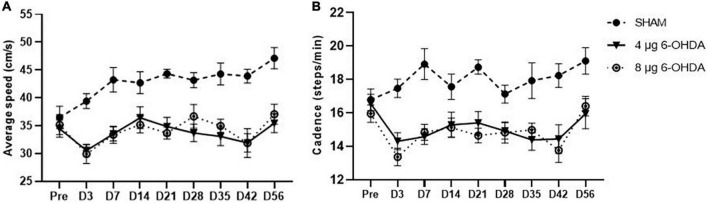
Time-course changes of the average speed **(A)** and cadence **(B)** in the gait analysis following 4-μg and 8-μg 6-OHDA injection. All results are presented as mean ± standard error of the mean (SEM).

A trend-effect of time × group (*F*_(16,238)_ = 1.53, *P* = 0.0890) and a significant effect of treatment (*F*_(2,30)_ = 29.46, *P* < 0.0001) were observed on the average speed (Figure 4A).

*Cadence*

Cadence is the number of steps per minute. A trend-effect of time × group (*F*_(16,238)_ = 1.50, *P* = 0.0880) and a significant effect of treatment (*F*_(2,30)_ = 24.26, *P* < 0.0001) were observed on the cadence (Figure 4B).

*Step cycle*

**FIGURE 5:**
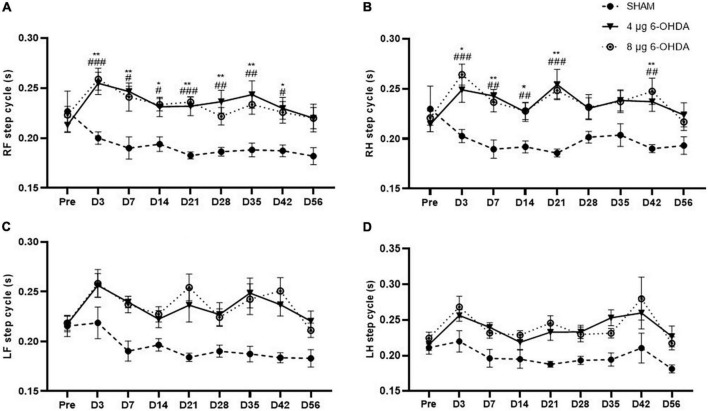
Time-course changes of the step cycle in RF **(A)**, RH **(B)**, LF **(C)**, and LH **(D)** paw in the gait analysis following 4-μg and 8-μg 6-OHDA injection. All results are presented as mean ± standard error of the mean (SEM). **P < 0.01, *P < 0.05, SHAM vs. 4-μg lesion group; ^####^*P* < 0.0001, ^###^*P* < 0.001, ^##^*P* < 0.01, ^#^*P* < 0.05, SHAM vs. 8-μg lesion group. RF, right front; RH, right hind; LF, left front; LH, left hind.

Step cycle (s) is the duration between two consecutive initial contacts of the same paw with the glass floor. Our data showed a significant effect of the group × time interaction on the RF (*F*_(16,238)_ = 1.79, *P* < 0.0001) and RH (*F*_(16,238)_ = 2.649, *P* < 0.001) paws for the step cycle parameter. *Post hoc* multiple comparisons showed significant differences in the RF step cycle of the 4 and 8 μg 6-OHDA lesion groups compared to the sham group on Days 3, 7, 14, 21, 28, 35, and 42 post-lesion (Figure 5A). Significant differences were also observed in the RH step cycle of the 4 and 8 μg 6-OHDA lesion groups on Days 3, 7, 14, 21, and 42 post-lesion (Figure 5B). There was no significant effect of group or time × treatment on the LF (*F*_(16,238)_ = 1.43, *P* = 0.1300) and LH (*F*_(16,238)_ = 0.80, *P* = 0.6814) paws for the step cycle parameter (Figures 5C,D).

*Stands and swings*

**FIGURE 6:**
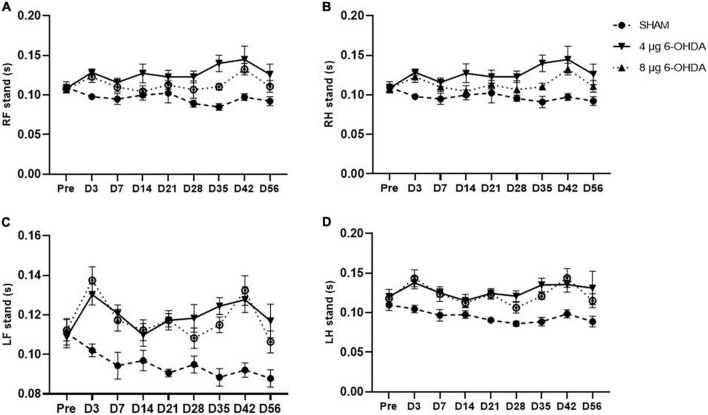
Time-course changes of the stands in RF **(A)**, RH **(B)**, LF **(C)**, and LH **(D)** paw in the gait analysis following 4-μg and 8-μg 6-OHDA injection. All results are presented as mean ± standard error of the mean (SEM). RF, right front; RH, right hind; LF, left front; LH, left hind.

**FIGURE 7:**
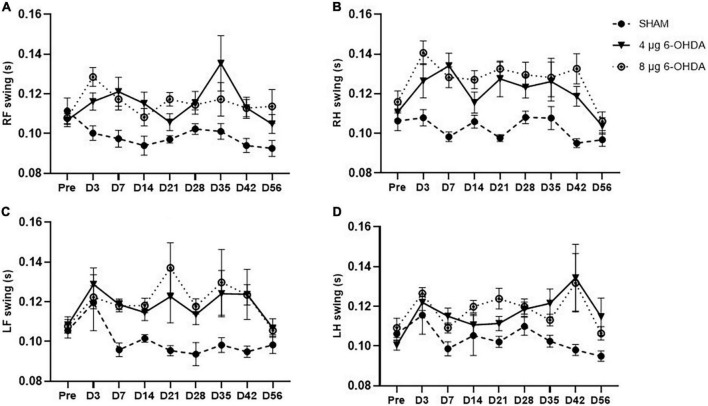
Time-course changes of the swings in RF **(A)**, RH **(B)**, LF **(C)**, and LH **(D)** paw in the gait analysis following 4-μg and 8-μg 6-OHDA injection. All results are presented as mean ± standard error of the mean (SEM). RF, right front; RH, right hind; LF, left front; LH, left hind.

The step cycle consists of two stages: the stand phase, which describes the duration of limb contact with the glass plate, and the swing phase, which describes the duration of limb no contact with the glass plate. While no group × time interaction was observed on any of the paws for the stands (Figures 6A–D) and swings (Figures 7A–D), a significant effect of treatment was independently observed on the RF stands (*F*_(2,30)_ = 11.04; *P* < 0.001), RH stands (*F*_(2,30)_ = 9.78; *P* < 0.001), LF stands (*F*_(2,30)_ = 18.91; *P* < 0.0001), and LH swings (*F*_(2,30)_ = 20.16; *P* < 0.0001). Similarly, a significant effect of treatment was independently observed on the RF stands (*F*_(2,30)_ = 19.36; *P* < 0.0001), RH stands (*F*_(2,30)_ = 40.83; *P* < 0.0001), LF stands (*F*_(2,30)_ = 11.76; *P* < 0.001), and LH swings (*F*_(2,30)_ = 8.16; *P* < 0.01).

4. Unilateral injection of 4 and 8 μg 6-OHDA induced contralateral rotation behavior in mice.

**FIGURE 8:**
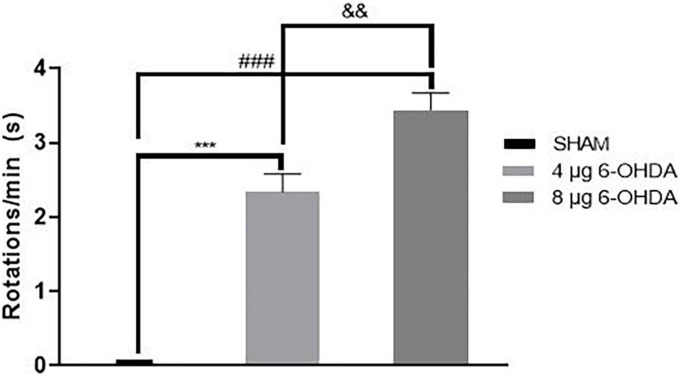
Contralateral turns in the apomorphine-induced rotation following 4-μg and 8-μg 6-OHDA injection. All results are presented as mean ± standard error of the mean (SEM). ***P < 0.001, SHAM vs. 4-μg lesion group; ^###^*P* < 0.001, SHAM vs. 8-μg lesion group. ^&&^*P* < 0.01, 4-μg lesion group vs. 8-μg lesion group.

When apomorphine is administered, animals that received 6-OHDA injection into the left striatum have asymmetrical rotation toward the contralateral side of the lesion. The SHAM group showed no significant rotational bias. The 4 and 8 μg 6-OHDA lesion groups had greater rotation numbers than the SHAM group (Supplementary material). Additionally, there was a significant difference between the 4 and 8 μg lesion groups (Figure 8).

5. Unilateral injection of 8 μg 6-OHDA induced impairment in motor coordination and balance on Day 43 post-lesion in mice.

**FIGURE 9:**
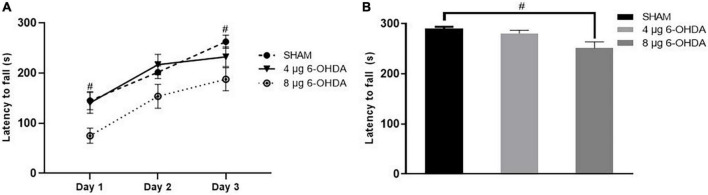
Latency to fall in the training phase **(A)** and test phase **(B)** in the rotarod test following 4-μg and 8-μg 6-OHDA injection. All results are presented as mean ± standard error of the mean (SEM). ^#^*P* < 0.05, SHAM vs. 8-μg lesion group.

In the training phase, the latency to fall of the 8 μg 6-OHDA lesion group decreased significantly compared to that of the sham group, and the difference in the 4 μg 6-OHDA lesion group failed to reach significance compared to that of the controls (Figure 9A). Similarly, in the test phase, a reduction in latency to fall was observed only in the 8 μg 6-OHDA lesion group (Figure 9B).

6. Unilateral injection of 4 and 8 μg 6-OHDA induced mild and intermediate loss of TH neurons in the SN and TH fibers in the striatum, respectively.

**FIGURE 10:**
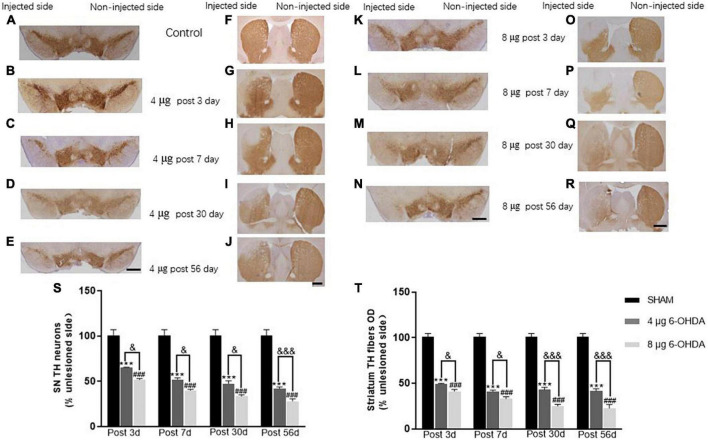
Representative images and quantification of TH immunostaining in SN **(A–E,K–N,S)** and striatum **(F–J,O–R,T)**. Scale bar: 0.5 mm for SN and 1 mm for striatum.

In the SN, a gradual loss of TH^+^ cells was observed in the 4 μg lesion group. After 3 days, 65.4 ± 0.11% TH^+^ cells remained compared to the non-injected side (Figures 10A,B), with a further decrease from 51.9 ± 2.00% (Figure 10C) after 7 days to 47.5 ± 3.07% after 30 days (Figure 10D). This remained stable over the duration of the rest of the study, with a slight decrease to 42.0 ± 4.17% at 56 days (Figure 10E). The 8 μg lesion group exhibited more loss of TH^+^ cells in the SN than the 4 μg lesion group. After 3 days, 51.7 ± 1.39% TH^+^ cells remained compared to the non-injected side (Figure 10K), with a further decrease to 40.0 ± 1.10% after 7 days (Figure 10L) and a subsequent slight decrease from 33.4 ± 1.80% after 30 days (Figure 10M) to 28.1 ± 2.46% after 56 days (Figure 10N).

A rapid loss of TH^+^ fibers was observed in the striatum following the unilateral injection of 4 μg 6-OHDA. After 3 days, 49.4 ± 5.48% TH^+^ fibers were present compared to the non-injected side (Figures 10F,G), with a further decrease to 41.1 ± 1.39% after 7 days (Figure 10H), and this remained stable over the duration of the rest of the study; 43.0 ± 2.36% after 30 days (Figure 10I) and 41.4 ± 0.25% (Figure 10J) after 56 days. Unilateral injection of 8 μg 6-OHDA induced a rapid loss of TH^+^ fibers in the striatum. After 3 days, 41.1 ± 2.03% TH^+^ fibers remained compared to the non-injected side (Figure 10O), with a further decrease from 33.0 ± 2.25% after 7 days (Figure 10P) to 24.9 ± 1.85% after 30 days (Figure 10Q), and this remained stable over the duration of the rest of the study, with slight decreases to 22.5 ± 3.98% after 56 days post-lesion (Figure 10R). In addition, there was a significant difference between the 4 and 8 μg lesion groups in the TH^+^ cells and TH^+^ fibers at different time points post-lesion (Figures 10S,T).

## Discussion

Animal models of PD play an important role in the discovery of potential therapies and molecular mechanisms of the illness (Dauer and Przedborski, 2003; Duty and Jenner, 2011; Im et al., 2016; Hamadjida et al., 2019; Salari and Bagheri, 2019; Rahman et al., 2020). The 6-OHDA striatal lesion model, more closely approximating early PD, has been commonly used for studying neuroprotective and neurotrophic strategies for PD, an active and important area of research (Lundblad et al., 2004; Marie et al., 2022; Mazzocchi et al., 2022). Here, we demonstrate that the intrastriatal injection of 4 and 8 μg 6-OHDA exerts spontaneous motor impairment, which partially recovered starting on Day 28 post-lesion. Forced motor impairment was induced in the 8 μg lesion group, which was stable on Day 43 post-lesion. In addition, we found that the 4 and 8 μg lesion groups displayed dose-dependent reductions in TH^+^ cells and TH^+^ fibers and caused dose-dependent apomorphine-induced rotation. The current study investigated the time-course changes in spontaneous motor deficits that occur following the intrastriatal delivery of 6-OHDA in mice, providing a better understanding of the behavioral characterization in the neurodegenerative process.

The cylinder test was used to measure spontaneous forelimb use, allowing the mice to start and stop movements freely (Glajch et al., 2012; Magno et al., 2019). Our results showed that both the 4 and 8 μg 6-OHDA lesion groups exhibited forelimb use asymmetry with a preference for the ipsilateral side on Day 3 and until Day 21 post-lesion, which is in agreement with a previous report showing that unilateral injections of 6-OHDA at doses of 3 μg (MFB) and 6 μg (striatum) elicited forelimb asymmetry on the contralateral side of the body (Lundblad et al., 2004). In addition, we found that the 4 and 8 μg lesion groups caused approximately the same degree of limb use asymmetry, although there was a significant difference between the 4 and 8 μg lesion groups in the TH^+^ cells and TH^+^ fibers at different time points post-lesion. Therefore, our studies showed that the cylinder test was not able to distinguish between lesions of different severities, which is in agreement with a previous study that reported that the cylinder test could not distinguish mild, intermediate, and severe lesion groups after the injection of 2.4 μg of 6-OHDA directly into the SN (Grealish et al., 2010). The time-course observation in the cylinder test showed that the 6-OHDA lesion group exhibited limb use asymmetry rapidly at 3 days post-lesion and partly recovered in the 4 μg lesion group starting on Day 28 and until Day 56 post-lesion. The 8 μg lesion group still exhibited forelimb asymmetry on Day 28 and 42 post-lesion and did not exhibit forelimb asymmetry on Day 56 post-lesion. Glajch et al. also reported that 6-OHDA injections in the MFB resulted in a significant decrease in the percentage of contralateral forelimb at 2, 4, and 6 weeks after surgery. In contrast, recovery of function was not found in this study, which may be associated with a near complete lesion (>90%) of nigrostriatal neurons induced by 6-OHDA injections in the MFB (Glajch et al., 2012). Supporting our results, partial recovery of behavioral parameters has been reported in the 6-OHDA lesion model (Stanic et al., 2003; Alvarez-Fischer et al., 2008). Alvarez-Fischer et al. (2008) reported that intrastriatal 4 μg 6-OHDA lesions in mice induced a behavioral impairment in the rotarod test after 3 days, and this motor performance slightly recovered after 7 days and made no difference compared to the control at 14 days post-surgery. Monville et al. (2006) also described the slight recovery in rotarod motor performance, but not of the MFB lesion, 6 weeks after induction by an intrastriatal lesion injection of 28 μg 6-OHDA. Truong et al. (2006) demonstrated that injections of 4, 8, and 16 g 6-OHDA into the MFB could produce behavioral deficits in rats, and similarly, this impairment normalized to baseline after weeks 8–10 post-surgery. Stanic et al. (2003) reported that rotational behavior was observed 2 weeks after the induction of intranigral 6-OHDA lesions in rats and this recovered by 16 weeks. Behavioral manifestation requires at least 50–60% loss of TH neurons, suggesting that compensatory processes play an important role in maintaining normal motor function (Brecknell et al., 1996; Sun et al., 2021). The spontaneous recovery of behavioral deficits may be related to compensatory mechanisms, such as striatal reinnervation, striatal D2 receptor upregulation, and increased activity of the remaining DA neurons (Finkelstein et al., 2000; Parish et al., 2001; Wang et al., 2022). An increase in DA turnover has been hypothesized to be a compensatory mechanism for dopaminergic neuronal loss in early PD stages (Sossi et al., 2002).

Gait disturbances are the most common symptoms of PD (Bange et al., 2022). Different patterns of gait impairments can be present throughout the progression of the disease (Mirelman et al., 2019; di Biase et al., 2020). It is known that gait abnormalities develop in the relatively late stages of PD (Ziégler, 2006; Lew, 2007). However, increasing evidence has shown that patients with early PD could also present with significant alterations in gait parameters such as speed, swing time, and cadence (Baltadjieva et al., 2006; Grajić et al., 2015). In the current study, the gait parameters were analyzed with time as a repeated measure, and average speed was added as a covariate in the dynamic paw parameters. Clinical studies have reported that slow walking speed is one of the earliest features of gait disturbances in PD (Chen et al., 2014; Kwon et al., 2017). Preclinical studies previously reported that walking speed decreased in the intrastriatal and MFB 6-OHDA lesion models in rats (Vlamings et al., 2007; Hsieh et al., 2011). Consistent with the above data, our results showed a trend toward a reduction in average speed, while no significant difference was found. One study showed a significant increase in cadence in the early stages of PD (Hausdorff et al., 2003). Conversely, the results of another study demonstrated that early moderate patients with PD showed a reduction in spatiotemporal gait parameters (speed and cadence) (Pistacchi et al., 2017). However, a significant reduction in cadence is observed in most PD animal models (Wang et al., 2012; Li et al., 2022). Our results showed that cadence declined, which is in accordance with those previously reported by Boix et al. (2018). In addition, the speed and cadence decreased rapidly in both the 4 and 8 μg 6-OHDA lesion groups 3 days post-lesion, which is well correlated with forelimb use asymmetry observed in the cylinder test. Our results showed a significant increase in RF and RH step cycles starting on Day 3 and until Day 42 post-lesion, indicating significant differences in the spatial gait parameters of the affected (contralateral) side of the lesioned mice. These results are in accordance with previous studies that have demonstrated gait asymmetry in patients with *de novo* PD (Grajić et al., 2015; Djurić-Jovičić et al., 2017). There were no significant changes in the swing and stand phases in the 6-OHDA lesion group, although an increasing trend was observed. The increase in the RF and RH step cycles slightly recovered 56 days post-lesion, which is well correlated with the recovery of motor impairment in the cylinder test. No significant differences were found in other parameters, such as print area and print width (data not shown), which correlated with the extent of TH neuronal loss in the SN and coincided with results previously reported showing that a moderate striatal lesion in rats did not change the print length and area of the animals (Boix et al., 2018). Similar to the cylinder test, our studies showed that gait analysis was not able to distinguish between lesions of different severities.

To determine if the unilateral 6-OHDA lesion results in forced movement deficits in mice, the rotarod test was used in which the animals were forced to initiate movement and continue to walk to avoid falling (Carter et al., 2001). During the training and test phase, the 8 μg 6-OHDA lesion group, but not the 4 μg lesion group, reduced the time on the accelerating rotarod at Day 43 post-lesion, suggesting a dose-dependent reduction of time in the forced movement. This result is consistent with a previous study showing that injection with increasing concentrations of 6-OHDA produced increasing severity of cell loss and behavioral changes (Truong et al., 2006). Our immunohistochemistry results also showed that the 8 μg 6-OHDA group induced a larger lesion than the 4 μg group. The accelerating rotarod test was able to distinguish between the two groups, which is in agreement with a previous study that observed that the accelerating protocol provides a more sensitive test to correlate motor deficits against lesion size (Iancu et al., 2005; Monville et al., 2006; Smith et al., 2012). Apomorphine-induced rotational behavior is a sensitive indicator of unilateral dopaminergic 6-OHDA lesioning to confirm DA cell loss in the SN (Brundin et al., 1986; Casas et al., 1988; Carman et al., 1991; Truong et al., 2006). Three weeks after 6-OHDA injection, highly significant rotational behavior was observed in both the 4 and 8 μg 6-OHDA lesion groups. Similar to the rotarod test, there is a 6-OHDA dose-dependent increase in the number of apomorphine-induced rotations, which is in agreement with a previous report showing that striatal lesions have a strong correlation between dopamine neuron loss and rotational asymmetry (Henderson et al., 2003; Henderson and Watson, 2003; Bagga et al., 2015; Su et al., 2018). Generally, the rotarod test and apomorphine-induced rotational test are able to distinguish between lesions of different severities and provide two useful tools for the assessment of functional recovery in the screening of novel potential therapies, which is in agreement with a previous study (Przedborski et al., 1995; Truong et al., 2006).

In the current study, the 4 μg lesion group induced 40–50% TH^+^ cells and TH^+^ fiber loss 3–7 days post-lesion, with a slight decrease to 60% after 56 days post-lesion. Generally, motor deficits require at least 50% loss of nigral dopaminergic neurons and striatal TH fibers in rats and mice (Gombash et al., 2013; Van der Perren et al., 2015). However, Truong et al. (2006) developed a graded rat model of PD and found that some significant changes could be observed even when cell loss in the SN was approximately 40%. In agreement with this study, we also observed significant behavioral changes in the cylinder test and gait analysis when approximately 40% TH^+^ cells were lost in the SN and 50% TH^+^ fibers were lost in the striatum at 3 days post-lesion. These results may be associated with the rapid loss of TH^+^ fibers in the striatum, yet the compensation mechanism has not been established immediately. These results are in total agreement with previous studies showing that the intrastriatal injection of 6-OHDA induced the same extent of nigrostriatal degeneration in mice (Lundblad et al., 2004; Wang et al., 2022).

## Conclusion

In summary, injection of 4 and 8 μg 6-OHDA induced dose-dependent lesions of the nigrostriatal system and corresponding behavioral consequences in mice. The spontaneous behavioral impairment was partially reversible over a 2-month time course in both groups. The forced movement impairment was still present in the 8 μg lesion group. The rotarod test and apomorphine-induced rotational test are able to determine lesion extent and provide two useful tools for the evaluation of future therapeutic strategies.

### Future perspectives

Therefore, future studies can use different doses of 6-OHDA to develop graded models of different (early, moderate, and advanced) clinical stages of PD in mice. Notably, the impairment induced by 6-OHDA was partially reversible over time, suggesting the possibility of potential compensatory mechanisms. This work highlights aspects of the behavioral characterization process in the 6-OHDA mouse model that can be applied to studies assessing novel potential therapies and unraveling molecular mechanisms of PD, with implications for the testing and timing of novel neuroprotective agents.

## Data availability statement

The original contributions presented in this study are included in the article/Supplementary material, further inquiries can be directed to the corresponding author.

## Ethics statement

The animal study was reviewed and approved by the Institutional Animal Care and Use Committee of the Institute of Laboratory Animal Sciences.

## Author contributions

CQ conceived and designed the experiments and acquired the funding. XS designed and performed the behavior tests and wrote the manuscript. XL performed the behavior tests. LZ, YZ, XQ, and SW performed the immunohistochemistry process. All authors contributed to the article and approved the submitted version.

## Abbreviations

6-OHDA: 6-hydroxydopamine
TH: tyrosine hydroxylase
PD: Parkinson’s disease
SN: substantia nigra.
